# Correction: Identifying key determinants and dynamics of SARS-CoV-2/ACE2 tight interaction

**DOI:** 10.1371/journal.pone.0259705

**Published:** 2021-11-03

**Authors:** Van A. Ngo, Ramesh K. Jha

Figs 1, 3, and 4 are incorrect; the publisher apologizes for the errors. The authors have provided corrected versions here.

**Fig 1:**
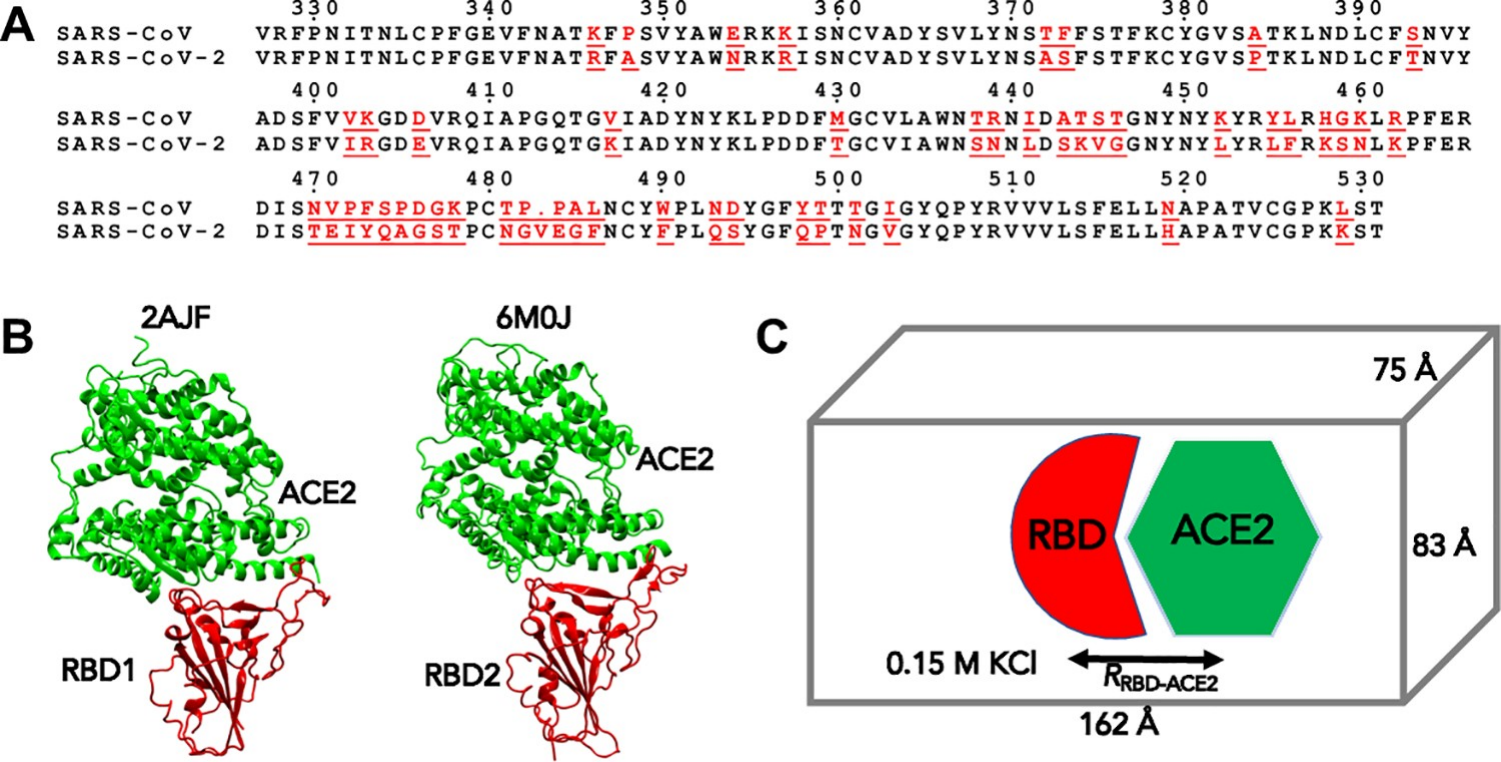
(A) Sequence alignment of receptor binding domains (RBD) of SARS-CoV and SARS-CoV-2. The residues underlined are mutations found in the RBDs. Any residue numbers referred in the text are positions in this sequence alignment. (B) X-ray structures of RBD1 of SARS-CoV (PDB: 2AJF) and RBD2 of SARS-CoV-2 (PDB: 6M0J), respectively, bound to human receptor angiotensin-converting enzyme 2 (ACE2). (C) MD simulation setup for RBD1-ACE2 and RBD2-ACE2 complexes.

**Fig 3:**
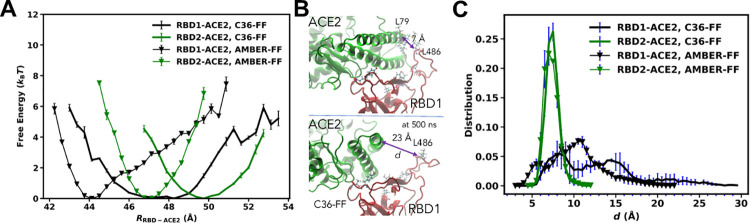
(A) Free-energy profiles computed as a function of the distance between the centers of mass of the RBDs and ACE2 using AMBER and C36 FFs. (B) Snapshots showing an initial configuration of RBD1-ACE complex and its configuration at 500 ns from the simulations using C36 FF. This 500 ns configuration was reproducible and showed in 3 out of 8 replicates (C) Distribution of the distance *d* between the Cαs of L486 of RBD1 and L79 of ACE2 and compared with *d* of F486-L79 in RBD2-ACE2.

**Fig 4:**
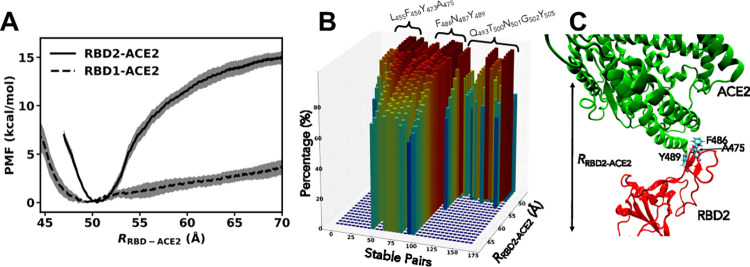
(A) Free-energy profiles computed from US simulations as function of *R*_RBD-ACE2_ using C36-FF. (B) Three-dimension distributions of the stable pairs (refer Fig 2) as function of the biasing distance between the centers of mass of RBD2 and ACE2. (C) A snapshot during the Umbrella simulations using *R*_RBD-ACE2_ = 70 Å. Residue A475 in RBD2 is located right behind F486.
