# Supplementary figures and images for: Identifying key determinants and dynamics of SARS-CoV-2/ACE2 tight interaction
Source: PLoS One. 2021 Sep 28;16(9):e0257905. doi: 10.1371/journal.pone.0257905 (PMC8478251; doi:10.1371/journal.pone.0257905)

S1 Fig.

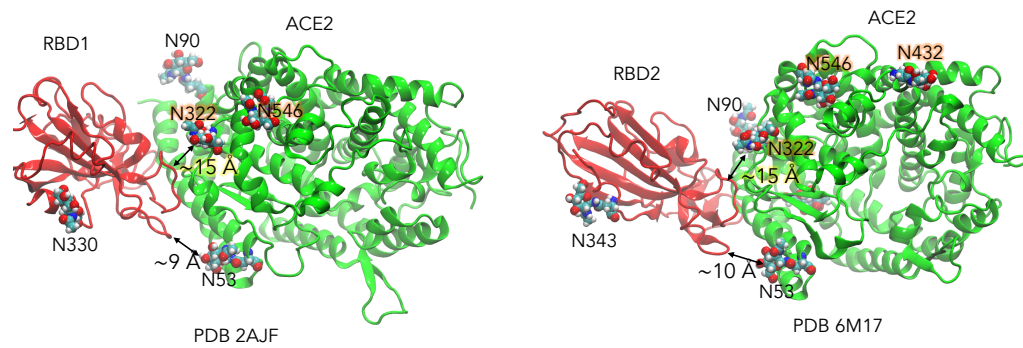

Supplement: S1 Fig — Snapshots from molecular dynamics simulations performed by the Shaw group (https://www.deshawresearch.com/downloads/download_trajectory_sarscov2.cgi/) showing distance of ACE2 N-glycans N322 and N53 from the nearest RBD1 (left, PDB code 2AJF) and the RBD2 (right, PDB code 6M17) residues. Other N-glycans on ACE2 and RBD are also shown and lie at a distance greater than 15 Å from the interface. (PDF) [file pone.0257905.s001.pdf]

S2 Fig.

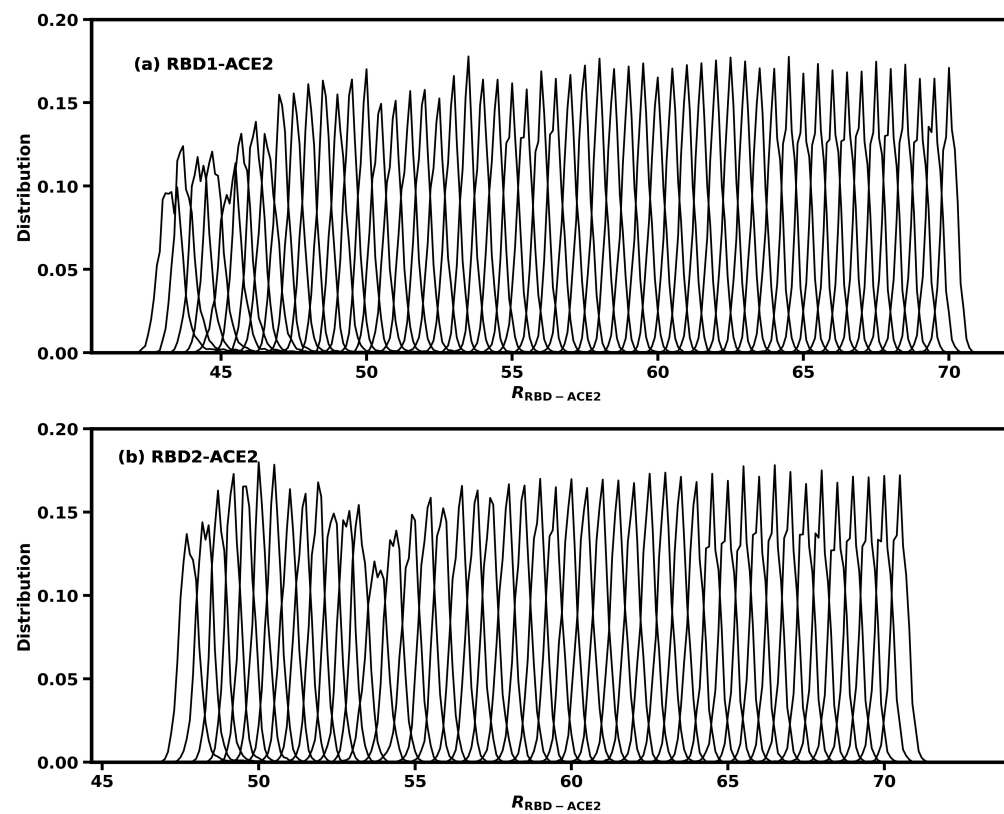

Supplement: S2 Fig — (PDF) [file pone.0257905.s002.pdf]

S3 Fig.

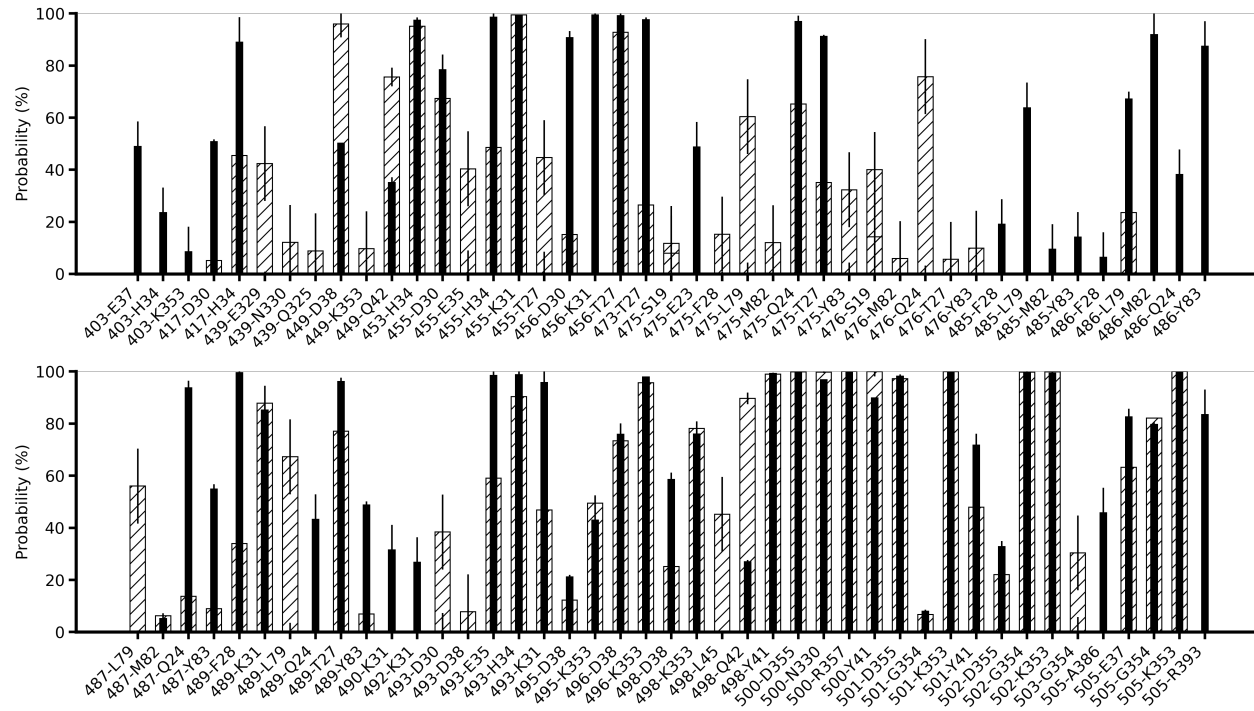

Supplement: S3 Fig — The first number corresponds to RBD based on alignment in Fig 1A. A stable pair is defined to have a probability larger than 60% during the simulation times. NOTE: S19 interactions are split into two parts that include the terminal group of residue-18 on ACE2. (PDF) [file pone.0257905.s003.pdf]

S4 Fig.

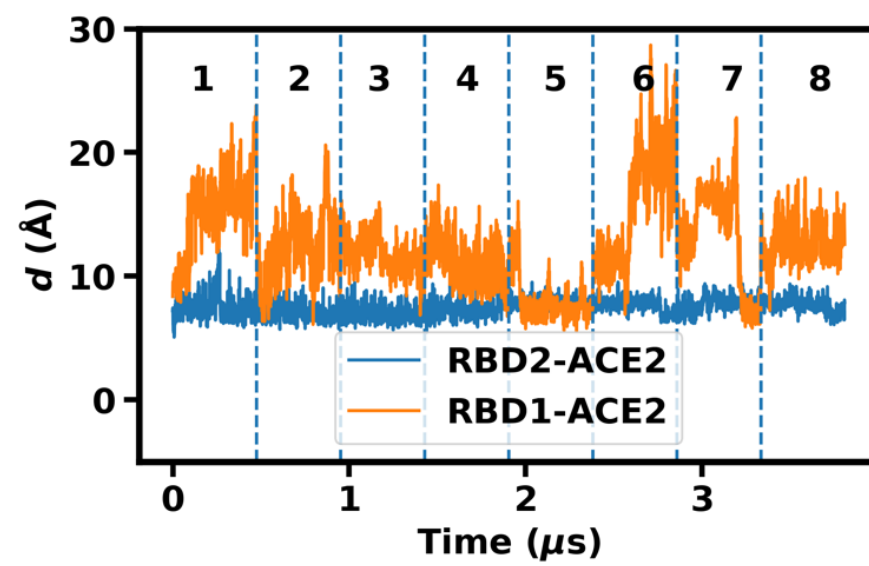

Supplement: S4 Fig — Dashed lines indicate the starting point of independent simulations with the indicated numbers. The value of d ≥ 23 (Fig 3B) occurs a few times in case of RBD1-ACE2. Overall, the interactions between L486 and L79 in RBD1-ACE2 complex is shown to be weaker than corresponding F486 and L79 in RBD2-ACE2. (PDF) [file pone.0257905.s004.pdf]

S5 Fig.

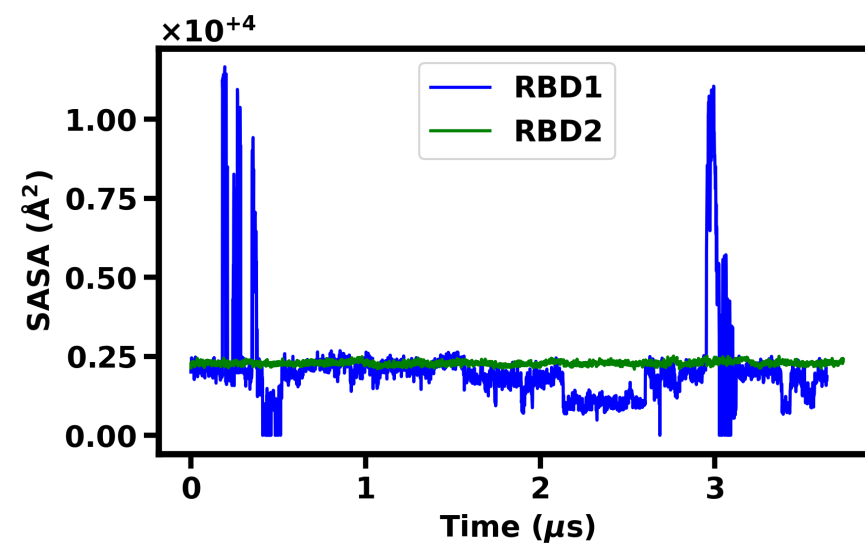

Supplement: S5 Fig — VMD [DOI:10.1016/0263-7855(96)00018-5] was used to compute SASA for the interface residues. A script can be found here: https://www.ks.uiuc.edu/Research/vmd/mailing_list/vmd-l/att-18670/sasa.tcl. (PDF) [file pone.0257905.s005.pdf]
