# Supplementary material for: Identifying key determinants and dynamics of SARS-CoV-2/ACE2 tight interaction
Source: PLoS One. 2021 Sep 28;16(9):e0257905. doi: 10.1371/journal.pone.0257905 (PMC8478251; doi:10.1371/journal.pone.0257905)
Supplement: Attachment — Submitted filename: Response_to_Reviewers.docx [file pone.0257905.s009.docx]

**Response to Reviewers**

Additional Editor Comments:

The reviewers have several important points that I am asking you to consider in a revised version. I understand that performing additional calculations is not always practical, and if you choose to not perform some of the calculations that the reviewers are suggesting (MMPBSA / SASA etc) , please justify in your answer why it is not necessary. Please consider the request from the reviewers to discuss your results in the context of recent publications, in particular these that address the role and importance of glycosylation of the protein.

 Reply: We put our best effort to address reviewer comments.

Journal Requirements:

When submitting your revision, we need you to address these additional requirements.

1. Please ensure that your manuscript meets PLOS ONE's style requirements, including those for file naming. The PLOS ONE style templates can be found at

https://journals.plos.org/plosone/s/file?id=wjVg/PLOSOne_formatting_sample_main_body.pdf and

<https://journals.plos.org/plosone/s/file?id=ba62/PLOSOne_formatting_sample_title_authors_affiliations.pdf>

Reply: We fixed the title page according to the guidelines.

2. Thank you for stating the following in the Acknowledgments Section of your manuscript:

[Research was supported by the DOE Office of Science through the National Virtual Biotechnology Laboratory, a consortium of DOE national laboratories focused on response to COVID-19, with funding provided by the Coronavirus CARES Act. V.A.N is a Director’s Postdoctoral Fellow at LANL and is partially funded by Laboratory Directed R&D Postdoctoral Research and Development fellowship (20170692PRD4). The work was authored under Triad National Security, LLC (“Triad”)Contract No. 38089233218CNA000001 with the U.S. Department of Energy. This research used computational resources provided by the Los Alamos National Laboratory Institutional Computing Program (under w20_foldamers to R.K.J. and x20_simcovid19 to V.A.N.), which is supported by the U.S. Department of Energy National Nuclear Security Administration under Contract No. 89233218CNA000001.]

We note that you have provided funding information that is not currently declared in your Funding Statement. However, funding information should not appear in the Acknowledgments section or other areas of your manuscript. We will only publish funding information present in the Funding Statement section of the online submission form.

Please remove any funding-related text from the manuscript and let us know how you would like to update your Funding Statement. Currently, your Funding Statement reads as follows:

 [Research was supported by the DOE Office of Science through the National Virtual Biotechnology Laboratory, a consortium of DOE national laboratories focused on response to COVID-19, with funding provided by the Coronavirus CARES Act.

The funders had no role in study design, data collection and analysis, decision to publish, or preparation of the manuscript.]

Reply: We moved the funding information from the Acknowledgement to Funding Statement

Additionally, because some of your funding information pertains to Triad National Security, LLC, we ask you to provide an updated Competing Interests statement, declaring all sources of commercial funding.

In your Competing Interests statement, please confirm that your commercial funding does not alter your adherence to PLOS ONE Editorial policies and criteria by including the following statement: "This does not alter our adherence to PLOS ONE policies on sharing data and materials.” as detailed online in our guide for authors  [http://journals.plos.org/plosone/s/competing-interests](about:blank).  If this statement is not true and your adherence to PLOS policies on sharing data and materials is altered, please explain how.

Please include your amended statements within your cover letter; we will change the online submission form on your behalf.

Reply: We provided the Competing Interests Statement as requested.

3. We note that you have included the phrase “data not shown” in your manuscript. Unfortunately, this does not meet our data sharing requirements. PLOS does not permit references to inaccessible data. We require that authors provide all relevant data within the paper, Supporting Information files, or in an acceptable, public repository. Please add a citation to support this phrase or upload the data that corresponds with these findings to a stable repository (such as Figshare or Dryad) and provide and URLs, DOIs, or accession numbers that may be used to access these data. Or, if the data are not a core part of the research being presented in your study, we ask that you remove the phrase that refers to these data.

Reply: in the revised version, we provided a specific number for the benchmark statement, namely “the same setup for the simulations using NAMD-GPU (timestep = 2 fs) can experience four times slower than AMBER-GPU (timestep = 4fs) on a test system of ~200k atoms, using NVIDIA Tesla P100 SXM2 GPGPUs”

4. Thank you for submitting the above manuscript to PLOS ONE. During our internal evaluation of the manuscript, we found significant text overlap between your submission and the following previously published works, some of which you are an author.

<https://www.cell.com/cell/fulltext/S0092-8674(20)30262-2?_returnURL=https%3A%2F%2Flinkinghub.elsevier.com%2Fretrieve%2Fpii%2FS0092867420302622%3Fshowall%3Dtrue>

We would like to make you aware that copying extracts from previous publications, especially outside the methods section, word-for-word is unacceptable. In addition, the reproduction of text from published reports has implications for the copyright that may apply to the publications.

Please revise the manuscript to rephrase the duplicated text, cite your sources, and provide details as to how the current manuscript advances on previous work. Please note that further consideration is dependent on the submission of a manuscript that addresses these concerns about the overlap in text with published work.

We will carefully review your manuscript upon resubmission, so please ensure that your revision is thorough.

Reply: The paper mentioned in the link was one of the first papers that talked about spike protein. We have cited this paper in our work. While we cannot negate the fact that our tone at some places could have been swayed by that paper, I assure you that there is no deliberate copying of text verbatim. **Please help us (direct us) to the right paragraphs where you have been able to detect any overlapping extracts, if that is possible.**

[Note: HTML markup is below. Please do not edit.]

Reviewers' comments:

Reviewer's Responses to Questions

**Comments to the Author**

1. Is the manuscript technically sound, and do the data support the conclusions?

The manuscript must describe a technically sound piece of scientific research with data that supports the conclusions. Experiments must have been conducted rigorously, with appropriate controls, replication, and sample sizes. The conclusions must be drawn appropriately based on the data presented.

Reviewer #1: Yes

Reviewer #2: Partly

2. Has the statistical analysis been performed appropriately and rigorously?

Reviewer #1: Yes

Reviewer #2: Yes

3. Have the authors made all data underlying the findings in their manuscript fully available?

The [PLOS Data policy](http://www.plosone.org/static/policies.action#sharing) requires authors to make all data underlying the findings described in their manuscript fully available without restriction, with rare exception (please refer to the Data Availability Statement in the manuscript PDF file). The data should be provided as part of the manuscript or its supporting information, or deposited to a public repository. For example, in addition to summary statistics, the data points behind means, medians and variance measures should be available. If there are restrictions on publicly sharing data—e.g. participant privacy or use of data from a third party—those must be specified.

Reviewer #1: Yes

Reviewer #2: No

4. Is the manuscript presented in an intelligible fashion and written in standard English?

PLOS ONE does not copyedit accepted manuscripts, so the language in submitted articles must be clear, correct, and unambiguous. Any typographical or grammatical errors should be corrected at revision, so please note any specific errors here.

Reviewer #1: Yes

Reviewer #2: Yes

5. Review Comments to the Author

Please use the space provided to explain your answers to the questions above. You may also include additional comments for the author, including concerns about dual publication, research ethics, or publication ethics. (Please upload your review as an attachment if it exceeds 20,000 characters)

Reviewer #1: 1) Authors have not included any glycans in their simulations. Recent papers have shown that some of these glycans are important for binding (e.g., glycan – glycan interactions on the RBD – ACE2 binding). For example:
https://www.biorxiv.org/content/10.1101/2021.03.30.437783v1
https://www.biorxiv.org/content/10.1101/2020.07.09.193680v1.full

Authors should consider explaining their results in light of these papers and if possible, include how their results compare to studies conducted with glycans included.

Authors should briefly talk about potentially affect in the conformational dynamics of the protein when glycans are removed which can indirectly affect interactions?

Reply: This is a very good point. When we initiated this work, there were only very limited number of structures of the RBD2-ACE2 complex available. Further, they showed discrepancy in their glycosylation pattern. We also found out in the literature that the protein expressed from a source, such as insect cells vs mammalian or human cells can perturb the glycosylation. Hence, in order to avoid biasing towards incorrect glycosylation and also since some of them were far from RBD2, we did not include them in our simulations. We have added this explanation in the Methods section.

“When building the models, we did not include glycans in either of the complexes. In the RBD1-ACE2 structure, the asparagine (N90) linked glycan (on ACE2), close to the interface, showed an extended form consisting of three sugar units (PDB: 2AJF). In RBD2-ACE2, N90 linked glycan showed a large discrepancy from structure to structure. While we saw only one sugar unit covalently attached to the N90 in two cases (PDBs: 6M0J and 7KMB), another structure of the RBD2-ACE2 complex (PDB: 6M17) showed two sugar units. Finally, the third form of glycosylation was also observed in a structure with three sugar units linked to N90 (PDB: 6VW1). Except for the latter one, in all RBD2-ACE2 complexes, the nearest amino acid to the glycan sugar T415 was >8 Å and expected to insignificantly contribute to the binding interaction. The structures of RBD-ACE2 complexes (PDB: 2AJF for RBD1-ACE2 & 6VW1 for RBD2-ACE2) that show long glycan on N90 also showed similar interaction with proximal residues T415 and R408 on RBD. Considering the two complexes were expressed using the same insect cell lines and 6VW1 experimental structure was determined by molecular replacement using 2AJF as structural template, we could not negate the effect of expression system on glycan pattern [20] and structural biases of solved structure towards the molecular replacement template. Further, mutation of N90 to eliminate glycosylation was shown to improve spike protein-mediated human cell infection [21], which is contrary to the observation based on interdomain contacts from the structure. Glycan linked to N322 on ACE2 also showed >8 Å distance from the nearest amino acid on the RBD. We also compared these dynamics modeled by the CHARMM force field with the model of the complexes using the AMBER force field in the presence of N-glycans. In the dynamics of the complexes using the AMBER force field, the N-glycan sidechains were at least 9 Å away from the residues of the RBD at the interfaces (**S1 Fig**). Due to the overall discrepancy in glycan from one structure to another and a low binding energy contribution expected from shorter glycan, the complexes were prepared in the absence of them.”

2) Authors report that the results differ between the CHARMM and AMBER forcefields but they do not discuss possible reasons behind this observation. The authors should consider explaining the reasons behind the reported differences. Additionally, while most of the analyses in the manuscript were done using both the CHARMM and AMBER forcefields, the authors used only the CHARMM forcefield for PMF and the reasons behind the change were not stated. Few sentences explaining the rationale behind this would be useful.

Reply: We added some discussions about the reasons of the difference between CHARMM and AMBER force field. One major reason is that both force fields use slightly different parametrization strategies (see DOI: [10.1002/jcc.20082](https://doi.org/10.1002/jcc.20082)). For instance, CHARMM and AMBER also use different approaches to compute charges for proteins: while CHARMM is based the QM calculations using HF/6-31G* for supramolecular data, AMBER is based on RESP (Restrained Electrostatic Potential) to fit to the QM calculations. A thorough comparison between AMBER and CHARMM can be found in the work by McKerell (DOI: [10.1002/jcc.20082](https://doi.org/10.1002/jcc.20082)). We used the D.E. Shaw’s simulation data with Amber forcefield (available in the link: <https://www.deshawresearch.com/downloads/download_trajectory_sarscov2.cgi/>). Our goal was to see if different force fields gave rise to different observation. We showed here that, in fact, different force fields yield differences in dynamics between RBD1/ACE2 and RBD2/ACE2 complexes (Fig 3). We only computed PMF for one force field for the complexes. We explained this in the main text, which reads “Since the comparison between the equilibrium simulations of RBD1/ACE2 and RBD2/ACE2 (Fig 3) yields similar differences for both force fields, it is reasonable to run umbrella sampling simulations for one force field, namely C36FF, to sample distances away from the global minima.”

3) Authors can consider performing MM-PBSA to get the binding energies and to also get the per residue energy decompositions.

Reply: MM-PBSA calculations and “the per residue energy decomposition” were computed in previous work, for instance, Zhang et al, (*In silico* binding profile characterization of SARS-CoV-2 spike protein and its mutants bound to human ACE2 receptor, Brief Bioinformatics, 2021 PMCID: PMC8194596). However, the published work did not report clear different dynamics happening at the interfaces. Hence, we focused our work around the dynamics of the protein-protein interaction.

4) Authors should consider calculating the number and lifetime of hydrogen bonds or hydrophobic interactions, etc., between ACE2-RBD1/2 as it would be more informative for interaction comparison.

Reply: This is a very good suggestion. However, these types of calculations have been reported elsewhere for shorter simulation timescales, for instance, see Ref. [2–5,8,11,12,14,15,36] in the text.. Since these calculations were computed mostly for shorter timescales, we only reported what have not been discussed: the difference dynamics at the interfaces during longer timescales.

5) Authors should define or explain what “bound” probability means in the result section. Was the probability just based on a distance cutoff? Or a hydrogen bond is being defined as interaction?

Reply: We removed “bound” from the text. This is simply the probability computed based on distance cutoff. The implication of this probability is explained in the next sentence; it reads “high probabilities indicate highly interactive pairs, while low probabilities indicate weakly interacting pairs.” Yes, a hydrogen bond is included in this probability.

6) Author should report some statistic so as to show the convergence of their simulations.

Reply: Thank you for bringing this up. It is possible that the convergence has not reached. We aim to examine the dynamics of the interfaces in “longer” simulations compared to the presented simulations, which could shed more definitive answers for the convergence. Currently that is beyond the scope of this manuscript.

7) Authors observed a minima shift of 2Å in Fig. 3, which seems very small relatively. Authors should explain the significance of these results in more detail.

Reply: We thank the reviewer for pointing this out. There are a couple of types of the free-energy minimum shifts in Figure 3A. The first one is a shift of about 1.5 Å between RBD1-ACE2 and RBD2-ACE2 in the same force field. This one is related to different sequences of RBD1 versus RBD2 and the movement of the loops shown in Figure 3B. The second shift is due to difference in force field, that is, AMBER versus CHARMM. In AMBER force field the distances between RBDs and ACE2 are 2 Å closer than those in CHARMM force field. This indicates relatively stronger attractions between RBDs and ACE2 in AMBER force field. Also, the shapes of the profiles are visually broader in CHARMM than in AMBER. We discussed this point in the text, which reads “When modeled with the C36 FF, the global free-energy profile of RBD1-ACE2 complex with a global minimum (at *R*_RBD-ACE2_ = 47-48.5Å) was broader than the RBD2-ACE2 complex that showed a global minimum (at *R*_RBD-ACE2_ ≈ 50 Å) (**Fig 3)**. This suggests that the RBD1-ACE2 interface is less tight than the RBD2-ACE2 interface.” (Pg 9, Ln 242-246).

8) Authors can also consider talking about water-mediated interactions at the interfaces.

Reply: Water-mediated interactions are manifested in the pair-residue interactions and the free-energy profiles in Fig. 3A. We are also more interested in the conformations of the loops of RBDs as shown in Figure 3B.

9) Authors have reported a lot of interesting results, but they need to provide more explanation on how it connects to the biology and physiology of the disease. Especially with respect to the current variants, they may also suggest some mutants which they believe can make the binding stronger in the future, hence making the variant more lethal.

Reply: We added a paragraph to include some of the observed mutations in the RBD of the spike protein in the new variants of SARS-CoV-2.

“During the completion of our work, newer variants of the SARS-CoV-2 evolved, that showed mutations in the spike protein including the RBD2. It is expected that some of them will perturb the interaction between ACE2 and spike protein either by creating new interactions, or by stabilizing the spike protein itself, as suggested from a recent published article [47]. Our finding showed N501 as an important residue of the cluster Q493/T500/N501/G502/Y505 that give binding advantage to RBD2 over RBD1 (**Fig 2**), but this cluster is also first to dissociate from ACE2 (**Fig 4B**) indicating sub optimal interactions compared to other clusters. N501 has been identified in several new variants to be mutated to tyrosine. Mutations like N501Y is expected to provide larger surface area for stabilizing the RBD2 or for interaction with ACE2. E484 and T478 were not identified as interfacial residues during our simulations, as they did not meet the cutoff distance. But new mutations such as E484K and T478K on RBD2 in evolved SARS-CoV-2 variants are expected to bring these positions within the interface cutoff due to longer side chain of lysine and hence perturb the binding interaction. Understanding these mutations becomes valuable not only to identify the transmissibility of the SARS variant but also whether any therapeutic or diagnostic measures get compromised.”

[47] Shorthouse D, Hall Benjamin A. SARS-CoV-2 Variants are Selecting for Spike Protein Mutations that Increase Protein Stability. Biophysics; 2021 Jun. doi:10.1101/2021.06.25.449882

Reviewer #2: Comments for the Authors

The outbreak and spread of COVID-19 diseases caused by the severe acute respiratory syndrome coronavirus 2 (SARS-CoV-2) infection now is well-known as a global concern to the public health worldwide. Angiotensin-converting enzyme 2 (ACE2) is an enzyme attached to the cell membranes of cells located in multiple tissues including lungs, arteries, heart, kidney, and intestines. It also serves as the entry point into cells for some coronaviruses, including severe acute respiratory syndrome coronavirus (SARS-CoV), and SARS-CoV-2. In this study, in order to pinpoint key residues involved in the SARS-CoV receptor binding domain (RBD, RBD1)-ACE2 and SARS-CoV-2 receptor binding domain (RBD, RBD2)-ACE2 binding, Ngo et al. performed microsecond simulations for these two complexes using different force fields together with free energy calculation and mutation studies, indicating that F486/N487/Y489/A475 is perhaps the most critical cluster of amino acids that appear to collectively induce strong interactions between RBD2 and ACE2. Taken together, the microsecond direct simulations and Umbrella Sampling (US) simulations by defining a range of center-of-mass distances between RBD and ACE2 results provide insightful information pertaining to these indispensable residues responsible for their binding and would promote our understanding and facilitate future therapeutics and diagnostics strategical improvement, considering that multiple SARS-CoV-2 mutant strains were prevalent with stronger infections recently.

There are several points described below,

1.Did the authors provided the Supporting Information Figures? I didn’t find Supporting Information Figures of 1-3. It severely affects my understanding/evaluation of the major analyses and conclusions described in the main text, such as the overlaps of adjacent US windows.

Reply: We’re sorry that the Supporting Information document was not obvious in our submission. We submitted the document earlier. We have a revised version now and named Plos_Supp_Info_final_1.3

2.Page 2, line 41, why was the “Spike protein” capitalized?

Reply: This was typographical error. We will put in lower case.

3.Page 3, line 68, Is “L486/N487/Y48/P475” a typo? According to the context and Fig1A, it should be “F486/N487/Y489/A475”.

Reply: Thanks for pointing this out. We fixed this.

4.There are several resolved complex structures of RBD1-ACE2 and RBD2-ACE2 as shown in Method section (page 4), do they have large differences? Why did the authors select 2AJF and 6M0J for the simulations?

Reply: At the start of this project, these two structures were the highest resolution structure available, and hence we used them for our work.

5.The authors considered Zn2+ ions when building the whole simulation system, which seems located at the binding interfaces. However, the influence of Zn2+ ions were not studied and discussed.

Reply: No, Zn2+ is not located at the interface. It is located near the center of ACE2 far away from the interface and actually stabilizing the interdomain interface of ACE2. Others have shown that Zn2+ only fluctuates minimally during the course of MD simulations (**DESRES-ANTON-10905033; https://www.deshawresearch.com/downloads/download_trajectory_sarscov2.cgi/).**

6.When computing the potential of mean force (PMF), the authors only considered the distance between the two centers of mass. However, it can be seen from the Fig. 3B that ACE2 and RBD1 also showed rotational motion, which can be measured by defining an additional angle parameter. Taking both the distance and angle into the free energy calculation would provide more meaningful and comprehensive results.

Reply: We appreciate the suggestions, but we did not sample for relative angles between the two proteins. That would be way too expensive for this simple study of the interfaces of RBDs and ACE2.

7.I suggest that the authors also include the analysis of solvent-accessible surface area (SASA) monitor over time, which is closely associated with the molecular contacts of the interfacial residues.

Reply: We thank you for suggesting this. We computed SASA as a function of time, shown in S5 Fig in the supporting information. The time-series of SASA also confirm that the RBD1-ACE2 interface is much more flexible than the RBD2-ACE2 interface.

**S5 Fig.** Solvent accessible surface area (SASA) computed during an accumulated time of approximately 4 μs (0.5 μs/trajectory) for the initial residues at the interfaces of RBDs-ACE2. VMD [DOI:10.1016/0263-7855(96)00018-5] was used to compute SASA for the interface residues. A script can be found in this link: https://www.ks.uiuc.edu/Research/vmd/mailing_list/vmd-l/att-18670/sasa.tcl

8.Amber FF model yielded a measurable stronger interface with higher number of stable pairs than the C36-FF model. Why? Is it mainly attributed to their charge or vdW parameter difference?

Reply: The major differences between AMBER and C36FF include both charges and VDW parameters. At the interfaces, there are very few salt-bridges; so it is reasonable to assume that the VDW interactions in AMBER are stronger at the interface. We discussed this point in the text (Pg 9, Ln 231-232)

9.I suggest the authors to use different colors for two force fields showing in the Fig. 3A/C as it is now quite difficult to tell which is which.

Reply: We changed the color to make the presentation better.

10.In page 10, lines 249-250. Where are results of the Pearson correlation coefficient calculations?

Reply: We provided the number (~0.2) for the Pearson correlation coefficient calculations.

11.In page 11, lines 338-339, “The dramatic difference in the dynamics of these residue clusters is only observed in MD simulations of beyond 500 ns, which many previous studies [2–5,8,11,12,14,15,36] did not report.” There should be a Figure to support this observation.

Reply: Figure 3B actually shows the snapshot at approximately 500 ns. In any event, we plot the time-series.

S4 Fig. Time series of the distance d between the Cαs of L486 of RBD1 and L79 of ACE2 and F486 of RBD2 and L79 of ACE2. Dashed lines indicate the starting point of independent simulations with the indicated numbers. The value of *d* ≥ 23 (Figure 3B) occurs a few times in case of RBD1-ACE2. Overall, the interactions between L486 and L79 in RBD1-ACE2 complex is shown to be weaker than corresponding F486 and L79 in RBD2-ACE2.

12.In Fig. 4A, which force field was used for this analysis? In Fig. 4B, the color bar should be provided.

Reply: We used CHARMM36. We replotted Fig. 4B with color bars.

6. PLOS authors have the option to publish the peer review history of their article ([what does this mean?](https://journals.plos.org/plosone/s/editorial-and-peer-review-process#loc-peer-review-history)). If published, this will include your full peer review and any attached files.


If you choose “no”, your identity will remain anonymous but your review may still be made public.


**Do you want your identity to be public for this peer review?** For information about this choice, including consent withdrawal, please see our [Privacy Policy](https://www.plos.org/privacy-policy).

Reviewer #1: No

Reviewer #2: No

[NOTE: If reviewer comments were submitted as an attachment file, they will be attached to this email and accessible via the submission site. Please log into your account, locate the manuscript record, and check for the action link "View Attachments". If this link does not appear, there are no attachment files.]

While revising your submission, please upload your figure files to the Preflight Analysis and Conversion Engine (PACE) digital diagnostic tool, <https://pacev2.apexcovantage.com/>. PACE helps ensure that figures meet PLOS requirements. To use PACE, you must first register as a user. Registration is free. Then, login and navigate to the UPLOAD tab, where you will find detailed instructions on how to use the tool. If you encounter any issues or have any questions when using PACE, please email PLOS at [figures@plos.org](mailto:figures@plos.org). Please note that Supporting Information files do not need this step.
