# Supplementary material for: Identifying key determinants and dynamics of SARS-CoV-2/ACE2 tight interaction
Source: PLoS One. 2021 Sep 28;16(9):e0257905. doi: 10.1371/journal.pone.0257905 (PMC8478251; doi:10.1371/journal.pone.0257905)
Supplement: Attachment — Submitted filename: Response_to_Reviewer_v4.docx [file pone.0257905.s010.docx]

Dear Editor, Jerome Baudry, PhD,

We would like to thank you for your consideration to publish our manuscript.

Below is our response to a comment from Reviewer #1, while Reviewer #2 has no other concerns.

Reviewer #1: The reviewer agrees that there are discrepancies in the glycosylation patterns, but not including any glycosylation in the structure does not address the issue. As it has been well established that glycosylation is a critical part of RBD-ACE2 complexes such that it affects the dynamics, more so in light of the paper: https://www.pnas.org/content/118/19/e2100425118, where they report that the glycans at positions N90 and N322 interact most strongly with the RBD and the N322 glycan of ACE2 interacts tightly with the spike RBD in 24 out of the 36 RBD–ACE2 complexes simulated, forming 5 to 10 interactions on average. With these published results, an un-glycosylated system provides little additional information. The authors mention that glycosylation occurs at a distance of 8-9Å and hence cannot influence that dynamics or binding, which is incorrect. There are long distance cross talks/interactions (i.e., Allostery) within proteins as well. On the other hand, the comparison between the FF is important and the study reports the importance of FF while running such simulations. Overall, the reviewer understands that this work was done earlier but with the findings available now, a biologically inaccurate model provides little insight into the SARS-Cov2 biology. However, the authors can consider focusing on FF comparison part of the paper rather than the biology. Alternatively, authors may compare their data with simulation from other groups (for example: https://doi.ccs.ornl.gov/ui/doi/98; https://doi.ccs.ornl.gov/ui/doi/92) to make more coherent biological conclusions.

Reply: We thank the reviewer for the valuable comment. Yes, our simulations did not include glycans as we depended only on the existing structural information for ACE2 during our work. We agree that in some computational studies (Mehdipour et al, PNAS, 2021, <https://www.pnas.org/content/118/19/e2100425118>), glycans have shown to be important for the interactions between RDB and ACE2. But we would also like to tell that other studies have failed to conclude any significant binding contribution from different forms of glycans on ACE2 with the spike protein (Nguyen et al, Viruses, 2021, <https://doi.org/10.3390/v13050927> ). Quoting directly from Nguyen et al, “Notably, we find that this RBD-ACE2 contact signature is not altered by the presence of different glycoforms, suggesting that RBD-ACE2 interaction is robust”.

While these studies took effort to go beyond the glycan information presented by different experimental structures (x-ray or cryo-EM), and performed simulations with a few mature, long and branched glycans (HT1 and HT2 by Mehdipour et al; MAN9 and FA2 glycans by Nguyen et al), the basis of selection of such glycosylation patterns for simulation is less intuitive or supported by experiments, and can become misleading on its own. A very recent work on comprehensive characterization of glycosylation on ACE2 (Shahjahan et al, Glycobiology, 2020, <https://doi.org/10.1093/glycob/cwaa101> ) showed a large distribution of glycosylation patterns at ACE2 N322 position. The most populated glycan on N322 was bi-antennary (two branches) and smaller than the glycans used in simulation by Mehdipour et al and Nguyen et al in their studies (both used tri-antennary and/or chemically more complex glycan). Such differences in size and chemical complexity are further expected to reduce the impact on RBD-ACE2 interaction (Fig. 1).

Fig. 1. Discrepancy in choosing glycans on Asn322 (or N322) on ACE2. (A) Sahjahan et al showed a wide distribution of glycans on N322, with bi-antennary form to be most populated on ACE2. Curly brackets mean ambiguity of the subunit at any position in the glycan. (B) Mehdipour et al used various forms of glycan on N322 and observed tri-antennary glycoforms producing interactions with spike protein. (C) Nguyen et al used two forms of glycans on N322 of ACE2 in their MD simulations and observed no significant change in binding to spike protein.

A more recent publication by Allen et al (J. Mol. Biol, 2021, <https://doi.org/10.1016/j.jmb.2020.166762> ) titled “Subtle influence of ACE2 glycan processing on SARS-COV-2 recognition” concluded from their experimental study that the ACE2 glycan may not influence SARS-CoV-2 binding. Directly quoting from the abstract, “We generated a panel of engineered ACE2 glycoforms which were analyzed by mass spectrometry to reveal the site-specific glycan modifications. We then probed the impact of ACE2 glycosylation on S binding and revealed a subtle sensitivity with hypersialylated or oligomannose-type glycans slightly impeding the inter- action. In contrast, deglycosylation of ACE2 did not influence SARS-CoV-2 binding. Overall, ACE2 glycosylation does not significantly influence viral spike binding. We suggest that any role of glycosylation in the pathobiology of SARS-CoV-2 will lie beyond its immediate impact of receptor glycosylation on virus binding.”

Hence, we expect our findings based on models that excluded glycans to be biophysically relevant.

We went ahead and cited the papers by Mehdipour et al, Nguyen et al and Allen et al in our manuscript, which will be useful for the readers to keep in mind while making conclusions from our study and applying to their own work. We also discuss this in the text (Conclusion section).

We checked out these data set <https://doi.ccs.ornl.gov/ui/doi/92>, which used CHARMM force field and <https://doi.ccs.ornl.gov/ui/doi/98>, which have many REMD simulations. We don’t know exactly what temperature ranges were used here. We believe that the REMD simulations of the RDB-ACE2 complex were also ran with CHARMM36 force field since there was the setup files with CHARMM36 for simulations run with Gromacs. If the simulations were run with GROMOS force field, we would love to examine them in our future studies.

We hope that our response to the comment is satisfactory and look forward to see our manuscript go to the next stage in the publication process. We are really thankful to both reviewers whose comments helped us probe further into the biophysics of spike protein – ACE2 interaction. Please, feel free to let us know any immediate deficiency in the manuscript which we can improve upon.

Your sincerely,

Ramesh Jha & Van A. Ngo	Dated: 09-05-2021
